# Supplementary material for: Multiplexed measurement of variant abundance and activity reveals VKOR topology, active site and human variant impact
Source: eLife. 2020 Sep 1;9:e58026. doi: 10.7554/eLife.58026 (PMC7462613; doi:10.7554/eLife.58026)
Supplement: Supplementary file 3. [file elife-58026-supp3.docx]

| **Position** | **Variant** | **Described in** |
| --- | --- | --- |
| **5** | W5* | (Oldenburg et al., 2004) |
| **26** | A26T | (Watzka et al., 2011) |
| **26** | A26P | (Bodin et al., 2008) |
| **27** | L27V | (Peoc’h et al., 2009) |
| **28** | H28Q | (Watzka et al., 2011) |
| **29** | V29L | (Oldenburg et al., 2004) |
| **34** | A34P | (Harrington et al., 2011) |
| **36** | D36Y | (Loebstein et al., 2007) |
| **36** | D36G | (Watzka et al., 2011) |
| **41** | A41S | (Rieder et al., 2005) |
| **45** | V45A | (Oldenburg et al., 2004) |
| **52** | S52W | (Watzka et al., 2011) |
| **52** | S52L | (Schmeits et al., 2010) |
| **54** | V54L | (Bodin et al., 2008; Harrington et al., 2008) |
| **56** | S56F | (Watzka et al., 2011) |
| **58** | R58G | (Oldenburg et al., 2004) |
| **59** | W59R | (Wilms et al., 2008) |
| **59** | W59L | (Watzka et al., 2011) |
| **59** | W59C | (Watzka et al., 2011) |
| **66** | V66M | (Oldenburg et al., 2004) |
| **66** | V66G | (Watzka et al., 2011) |
| **68** | H68Y | (Osman et al., 2006) |
| **71** | G71A | (Watzka et al., 2011) |
| **77** | N77Y | (Watzka et al., 2011) |
| **77** | N77S | (Watzka et al., 2011) |
| **123** | I123N | (Watzka et al., 2011) |
| **128** | L128R | (Oldenburg et al., 2004) |
| **139** | Y139H | (Watzka et al., 2011) |

**References**

Bodin L, Perdu J, Diry M, Horellou M-H, Loriot M-A. 2008. Multiple genetic alterations in vitamin K epoxide reductase complex subunit 1 gene (VKORC1) can explain the high dose requirement during oral anticoagulation in humans. *J Thromb Haemost* **6**:1436–1439. doi:10.1111/j.1538-7836.2008.03049.x

Harrington DJ, Siddiq S, Allford SL, Shearer MJ, Mumford AD. 2011. More on: endoplasmic reticulum loop VKORC1 substitutions cause warfarin resistance but do not diminish gamma-carboxylation of the vitamin K-dependent coagulation factors. *J Thromb Haemost* **9**:1093–1095. doi:10.1111/j.1538-7836.2011.04249.x

Loebstein R, Dvoskin I, Halkin H, Vecsler M, Lubetsky A, Rechavi G, Amariglio N, Cohen Y, Ken-Dror G, Almog S, Gak E. 2007. A coding VKORC1 Asp36Tyr polymorphism predisposes to warfarin resistance. *Blood* **109**:2477–2480. doi:10.1182/blood-2006-08-038984

Oldenburg J, Rost S, Fregin A, Geisen C, Ivaskevicius V, Seifried E, Scharrer I, Heistinger M, Tuddenham E, Muller-Reible C, Zieger B. 2004. Mutations in the VKORC1 Gene Cause Warfarin Resistance, Warfarin Sensitivity and Combined Deficiency of Vitamin K Dependent Coagulation Factors. *Blood* **104**:277–277.

Osman A, Enström C, Arbring K, Söderkvist P, Lindahl TL. 2006. Main haplotypes and mutational analysis of vitamin K epoxide reductase (VKORC1) in a Swedish population: a retrospective analysis of case records. *J Thromb Haemost* **4**:1723–1729. doi:10.1111/j.1538-7836.2006.02039.x

Peoc’h K, Pruvot S, Gourmel C, dit Sollier CB, Drouet L. 2009. A new VKORC1 mutation leading to an isolated resistance to fluindione. *Br J Haematol* **145**:841–843. doi:10.1111/j.1365-2141.2009.07687.x

Rieder MJ, Reiner AP, Gage BF, Nickerson DA, Eby CS, McLeod HL, Blough DK, Thummel KE, Veenstra DL, Rettie AE. 2005. Effect of VKORC1 Haplotypes on Transcriptional Regulation and Warfarin Dose. *N Engl J Med* **352**:2285–2293. doi:10.1056/NEJMoa044503

Schmeits PCJ, Hermans MHA, van Geest-Daalderop JHH, Poodt J, de Sauvage Nolting PRW, Conemans JMH. 2010. VKORC1 mutations in patients with partial resistance to phenprocoumon. *Br J Haematol* **148**:955–957. doi:10.1111/j.1365-2141.2009.08017.x

Watzka M, Geisen C, Bevans CG, Sittinger K, Spohn G, Rost S, Seifried E, Müller CR, Oldenburg J. 2011. Thirteen novel VKORC1 mutations associated with oral anticoagulant resistance: insights into improved patient diagnosis and treatment. *J Thromb Haemost* **9**:109–118. doi:10.1111/j.1538-7836.2010.04095.x

Wilms EB, Touw DJ, Conemans JMH, Veldkamp R, Hermans M. 2008. A new VKORC1 allelic variant (p.Trp59Arg) in a patient with partial resistance to acenocoumarol and phenprocoumon. *J Thromb Haemost* **6**:1224–1226. doi:10.1111/j.1538-7836.2008.02975.x
