# Supplementary material for: Multiplexed measurement of variant abundance and activity reveals VKOR topology, active site and human variant impact
Source: eLife. 2020 Sep 1;9:e58026. doi: 10.7554/eLife.58026 (PMC7462613; doi:10.7554/eLife.58026)
Supplement: Peer review history

# Decision letter

**DOI:** 10.7554/eLife.58026.sa1

**Affiliations:** Weizmann Institute of ScienceIsrael; Weizmann Institute of ScienceIsrael; Washington University at St LouisUnited States

In the interests of transparency, eLife publishes the most substantive revision requests and the accompanying author responses.

**Acceptance summary:**

Chiasson et al. carry out a thorough experimental/computational analysis of the vitamin K epoxide reductase (VKOR) membrane protein. Using deep mutational scanning and structural bioinformatics analyses, they make several structural and functional inferences, for example, on the protein's exact topology, its functional site and positions that may be critical for folding versus activity. The work shows a clear path forward to get detailed and reliable structure/activity relationships in membrane proteins that defy experimental structure determination.

**Decision letter after peer review:**

Thank you for submitting your article "Multiplexed measurement of variant abundance and activity reveals VKOR topology, active site and human variant impact" for consideration by *eLife*. Your article has been reviewed by three peer reviewers, including Sarel Jacob Fleishman as the Reviewing Editor and Reviewer #1, and the evaluation has been overseen by John Kuriyan as the Senior Editor. The following individual involved in review of your submission has agreed to reveal their identity: Weikai Li (Reviewer #2).

The reviewers have discussed the reviews with one another and the Reviewing Editor has drafted this decision to help you prepare a revised submission.

We would like to draw your attention to changes in our revision policy that we have made in response to COVID-19 (https://elifesciences.org/articles/57162). Specifically, we are asking editors to accept without delay manuscripts, like yours, that they judge can stand as *eLife* papers without additional data, even if they feel that they would make the manuscript stronger. Thus the revisions requested below only address clarity and presentation.

Summary:

Chiasson et al. carry out a comprehensive experimental/computational analysis of the vitamin K epoxide reductase (VKOR) membrane protein. Despite being a major drug target, little is known about this protein's structure. Using deep mutational scanning and structural bioinformatics analyses, they make several structural and functional inferences, for example, on the protein's topology, its functional site and positions that may be critical for folding versus activity. The relative abundance of the mutations helps to interpret those causing warfarin sensitivity and clotting deficiency. Overall, these conclusions are well supported by the massive data which afford a thorough assessment of the mutational space. For instance, the authors propose that the clinically observed warfarin resistance due to VKOR polymorphisms is likely to be due to differences in warfarin binding rather than to protein abundance or activity. To conduct this study, the authors developed reporters to measure both the protein abundance and its activity providing a much more informative dataset than most studies that employ deep mutational scanning on membrane proteins. In summary, the study leverages large-scale functional data generated by the parallel multiplex assays to go beyond what a purely structural modeling study could achieve. The authors also provide a very detailed Materials and methods section that will be a valuable resource for future studies using multiplexed functional analysis of genetic variants.

Essential revisions:

Topology inferences:

1) In the evolutionary-coupling analysis, the authors used sequences from both prokaryotes and eukaryotes but they mention previously that the topology of the bacterial proteins could have been different from the eukaryotic ones. Can the authors provide evidence that the EVcouplings analysis is not biased to bacterial sequences, for instance by excluding them? If this analysis cannot be carried out, then please state how many bacterial sequences were used and perhaps add a caveat that this analysis may be biased towards the topology observed in bacteria (the work provides ample evidence from other analyses that the four TMD topology is correct).

2) While the large body of abundance, activity and evolutionary coupling data (Figure 3) is consistent with a four-helix topology, the authors do not explicitly rule out the possibility that TM2 is re-entrant (as suggested by hydropathy patterns predicted by the server octopus), and thus inverting the topology of the last two TMs as in the 3-TM model. The overall demonstration of the four-helix topology would be more convincing if a three TM model were shown to heavily violate some of the functional and coupling data.

3) The authors use the Hessa scale to compute helix-insertion energies but this scale is known to yield relatively high, even positive, energies to known TM domains, as the authors find here for three of four TMDs. Out of curiosity, I ran the human VKOR sequence in TopGraph, which uses the Elazar insertion energetics, and got four TMDs that match the authors' four quite closely and all insertion energies are negative. It may therefore be useful to include additional topology calculators.

4) To further support their conclusion, could the authors provide a comparison of abundance/activity to a hydropathy plot, and/or a comparison of hydropathy and lipophilicity (that can be calculated using the LIPS server) between VKOR homologs to support that the 4 TM topology is conserved between bacteria and mammals?

5) Introduction, second paragraph, when referring to the previous topology studies, Shen et al., 2017, provides a more definitive answer than those cited via looking at the disulfide bond pattern of human VKOR.

Structure modeling:

6) The authors used both I-TASSER and evfold. Why was this necessary? And, can the authors comment on the differences between the two models as well as provide the EVFold model in the supplement in addition to the I-TASSER one? Not surprisingly, the I-TASSER model seems heavily biased towards the 4 TM bacterial structure which is used as a starting template in the modeling simulations. The pre-TM2 loop that is not present in the bacterial structure contains some of the warfarin resistance mutants, so those residues might play a larger role in scaffolding the binding site, instead of forming the loop 'appendage' in the I-TASSER model. Do the evolutionary and I-TASSER models significantly differ in that region?

Localisation:

7) The authors mentioned that they did not directly measure localization of the VKOR variants, but didn't discuss whether this represents a limitation or not. To what extent would the abundance and activity data be affected by changes in trafficking and localization, for mutations in the ER localization sequences or elsewhere? Obviously, the localization of 2'695 (or even 697) variants could not be checked. But could the authors measure it for a representative subset of each cluster group with characteristic mutational pattern effects on abundance (Figure 4B)?

8) More generally, while the authors briefly mentioned the limitations of the approach (Discussion, third paragraph), they should make an effort to discuss them in the context of the distinct assays and data interpretation. How would the findings and conclusions of the study differ if these technical limitations could be addressed?

Additional comments:

1) There should be a control that correlates the GFP signal of mutant/truncation (e.g., those in Figure 1B) with the VKOR protein level (by western) to support that one can reliable deduce the relative abundance level from GFP signal for membrane proteins. In addition, in the text, it should be clarified whether N- or C- GFP is used in the mass abundance measurements and whether this makes any difference. The problem is that N- and C-GFP fused to a membrane protein have different effects. The C-GFP is a better reporter because if the N-terminal membrane protein is not folded properly, and the C-GFP may not be either. On the other hand, the N-GFP may be folded disregarding the misfolding of the C-terminal membrane protein. In addition, it is known that in mammalian cells the C-GFP may show fluorescence even the membrane protein is not properly folded.

2) Figure 1A TMD-del. The GFP should be on the ER luminal side.

3) There should be a representative supplementary figure showing the error bar of the specific activity (activity/abundance). It will be good to get a general feeling about how reliable this number is with the mass measurements. That said, in Figure 5 the overall conclusion looks quite convincing. The residues showing large changes should be labeled in Figure 5.

4) The abundance level of resistant mutations only changes by 2-3 fold in Figure 6C. It seems not a surprise that abundance is not causative of resistance because strong resistant mutations generally show several to hundred-fold of difference in IC50. The Discussion may benefit from some rewording.

# Reply: Author response

**DOI:** 10.7554/eLife.58026.sa2

Essential revisions:

Topology inferences:

1) In the evolutionary-coupling analysis, the authors used sequences from both prokaryotes and eukaryotes but they mention previously that the topology of the bacterial proteins could have been different from the eukaryotic ones. Can the authors provide evidence that the EVcouplings analysis is not biased to bacterial sequences, for instance by excluding them? If this analysis cannot be carried out, then please state how many bacterial sequences were used and perhaps add a caveat that this analysis may be biased towards the topology observed in bacteria (the work provides ample evidence from other analyses that the four TMD topology is correct).

Couplings from eukaryotic sequences alone show 4TM topology. Figure 3—figure supplement 2 shows the couplings-topology analysis repeated using only eukaryote sequences.

Residues coupled in the eukaryote alignment include anti-parallel contacts between TMD1-TMD2 and TMD1-TMD4 that are unique to the four TM helix topology, and inconsistent with a three TM topology. See Figure 3—figure supplement 2.

Thus, models made with and without bacterial sequences show a 4TM topology. To provide the best possible model in the main text, we included all sequences: 1,118 eukaryote, 5,731 bacterial, and 61 from environmental samples and viruses. We have added text detailing the composition of the full alignment, and the eukaryote-only results to the subsection “Human VKOR has four transmembrane domains”.

2) While the large body of abundance, activity and evolutionary coupling data (Figure 3) is consistent with a four-helix topology, the authors do not explicitly rule out the possibility that TM2 is re-entrant (as suggested by hydropathy patterns predicted by the server octopus), and thus inverting the topology of the last two TMs as in the 3-TM model. The overall demonstration of the four-helix topology would be more convincing if a three TM model were shown to heavily violate some of the functional and coupling data.

A 3-TM model is inconsistent with the couplings data (see answer to comment above, Figure 3—figure supplement 2). The evidence also rules out TMD2 being re-entrant (exiting on the same side of the membrane, rather than the opposite): antiparallel couplings show contacts between the full length of TMD1 and TMD2. Thus, TMD2 must pass fully through the membrane.

We have now more clearly explained how the evolutionary couplings are inconsistent with 3-TM topology in the subsection “Human VKOR has four transmembrane domains”.

3) The authors use the Hessa scale to compute helix-insertion energies but this scale is known to yield relatively high, even positive, energies to known TM domains, as the authors find here for three of four TMDs. Out of curiosity, I ran the human VKOR sequence in TopGraph, which uses the Elazar insertion energetics, and got four TMDs that match the authors' four quite closely and all insertion energies are negative. It may therefore be useful to include additional topology calculators.

We ran the human VKOR sequence in TopGraph with constraints that the N- and C- terminus have to both lie in the cytoplasm, and indeed it predicts four TMDs with negative insertion energies. We note that the main argument we use insertion energies to make still holds: TMD3 has a much more favorable insertion energy than the other TMDs according to TopGraph (TMD1: -6.3, TMD2: -5.5, TMD3: -12.6, TMD4: -4.3).

We’ve added text to the subsection “Human VKOR has four transmembrane”, that describe the TopGraph results.

4) To further support their conclusion, could the authors provide a comparison of abundance/activity to a hydropathy plot, and/or a comparison of hydropathy and lipophilicity (that can be calculated using the LIPS server) between VKOR homologs to support that the 4 TM topology is conserved between bacteria and mammals?

We used the AlignMe server (Stamm et al., 2014) to compute hydrophobicity of a bacterial VKOR homology MSA and a mammalian VKOR homolog MSA. We saw high concordance between values for both, supporting our conclusion that a four transmembrane topology is conserved between bacteria and mammals. We added the hydropathy plot as Figure 3—figure​ supplement 3B and added text explaining this result to the subsection “Human VKOR has four transmembrane domains”.

5) Introduction, second paragraph, when referring to the previous topology studies, Shen et al., 2017, provides a more definitive answer than those cited via looking at the disulfide bond pattern of human VKOR.

We agree and have added the Shen et al. citation to the Introduction.

Structure modeling:

6) The authors used both I-TASSER and evfold. Why was this necessary? And, can the authors comment on the differences between the two models as well as provide the EVFold model in the supplement in addition to the I-TASSER one? Not surprisingly, the I-TASSER model seems heavily biased towards the 4 TM bacterial structure which is used as a starting template in the modeling simulations. The pre-TM2 loop that is not present in the bacterial structure contains some of the warfarin resistance mutants, so those residues might play a larger role in scaffolding the binding site, instead of forming the loop 'appendage' in the I-TASSER model. Do the evolutionary and I-TASSER models significantly differ in that region?

The ITASSER model uses threading on the bacterial VKOR, whereas the EVcouplings model is folded by minimizing distance between coupled residues under a simple forcefield and no template structure. Therefore the ITASSER model may represent a more precise fold in places where there are few couplings or minimizing distances is a weak approximation. But the EVcouplings model may be more accurate where human VKOR is dissimilar to the bacterial. All in all, the ITASSER relies on the hypothesis that human VKOR has the same features as bacterial VKOR which has the potential to be more accurate because the template model has atomic resolution. We have confidence in that model in part because of its similarity to the EVcouplings results.

We examined how similar structurally the models were. Including all residues, RMSD is 6.556 Å.​ However, if we exclude the ER lumenal loop (residues 37-78), RMSD is 2.911 Å. The ER loop is largely unstructured and flexible, with the exception of a half helix that is hypothesized to run from positions 52 to 57. This half helix is only present in the ITASSER model; it is absent in our EVcouplings model. Beyond this, generally we observe that the ER loop in the EVcouplings model runs around the periphery of the protein, while the ITASSER model’s ER loop traverses the central cavity of the protein. Both models could be correct; it is most likely that the ER loop is dynamic, as it is involved in the transfer of electrons to the active site but also must allow for vitamin K to dock in the central cavity of the protein. While this ER loop contributes to a large RMSD overall, it most likely is reflecting dynamic biological states.

We have provided both EVcouplings model and the ITASSER model as Supplementary files 1 and 2, respectively, for readers to compare.

Localisation:

7) The authors mentioned that they did not directly measure localization of the VKOR variants, but didn't discuss whether this represents a limitation or not. To what extent would the abundance and activity data be affected by changes in trafficking and localization, for mutations in the ER localization sequences or elsewhere? Obviously, the localization of 2'695 (or even 697) variants could not be checked. But could the authors measure it for a representative subset of each cluster group with characteristic mutational pattern effects on abundance (Figure 4B)?

While we also see the value in performing these localization experiments, unfortunately we cannot conduct these follow-up experiments due to current circumstances.

8) More generally, while the authors briefly mentioned the limitations of the approach (Discussion, third paragraph), they should make an effort to discuss them in the context of the distinct assays and data interpretation. How would the findings and conclusions of the study differ if these technical limitations could be addressed?

This is a good point, so we have expanded this section to further comment on how these limitations have affected data and the conclusions we draw from them (Discussion).

Additional comments:

1) There should be a control that correlates the GFP signal of mutant/truncation (e.g., those in Figure 1B) with the VKOR protein level (by western) to support that one can reliable deduce the relative abundance level from GFP signal for membrane proteins. In addition, in the text, it should be clarified whether N- or C- GFP is used in the mass abundance measurements and whether this makes any difference. The problem is that N- and C-GFP fused to a membrane protein have different effects. The C-GFP is a better reporter because if the N-terminal membrane protein is not folded properly, and the C-GFP may not be either. On the other hand, the N-GFP may be folded disregarding the misfolding of the C-terminal membrane protein. In addition, it is known that in mammalian cells the C-GFP may show fluorescence even the membrane protein is not properly folded.

We agree that this is an important issue. Thus, we have included a new western blot, along with a plot showing strong correlation (R = 0.87) b​etween the abundance score and the intensity of GFP band normalized by loading control, as Figure 1—figure supplement 2.

2) Figure 1A TMD-del. The GFP should be on the ER luminal side.

We updated the schematic to show that GFP is in the lumen of the ER.

3) There should be a representative supplementary figure showing the error bar of the specific activity (activity/abundance). It will be good to get a general feeling about how reliable this number is with the mass measurements. That said, in Figure 5 the overall conclusion looks quite convincing. The residues showing large changes should be labeled in Figure 5.

We’ve added two panels in Figure 5—figure supplement 1 to address these points. Panel E shows specific activity score with error for a subset of variants, showing that error is small enough to distinguish between high and low ratio variants. In panel F, we present the histogram of specific activity coefficient of variation (CV), with 72% of variants showing CVs less than 0.25. We have also added more labels to Figure 5 to orient the reader to what residues are being shown as our experimentally determined active site.

4) The abundance level of resistant mutations only changes by 2-3 fold in Figure 6C. It seems not a surprise that abundance is not causative of resistance because strong resistant mutations generally show several to hundred-fold of difference in IC50. The Discussion may benefit from some rewording.

This is a good point, the level of resistance is something to consider, as well. To address this, we added text in the Discussion about the magnitude of resistance relative to protein abundance changes, and added a citation to Shen et al., 2018, which has data showing that the IC50s of many of these variants is much higher than wildtype.
